# Pulmonary alveolar proteinosis and first successful whole lung lavage in Sri Lanka: a case report

**DOI:** 10.1186/s13256-017-1218-2

**Published:** 2017-03-08

**Authors:** Janith Galhenage, Buddhika Weerasinghe, Wadasinghe Dilesha, Roshana Constantine, Bandu Gunasena

**Affiliations:** 1 Department of Respiratory Medicine, National Hospital for Respiratory Diseases, Welisara, Sri Lanka; 2 Department of Pathology, National Hospital for Respiratory Diseases, Welisara, Sri Lanka

**Keywords:** Whole lung lavage, Pulmonary alveolar proteinosis, Bronchoalveolar lavage, Case report

## Abstract

**Background:**

Pulmonary alveolar proteinosis is a rare disease characterized by accumulation of lipoproteinaceous material within alveoli. There are three clinically distinct forms: congenital, acquired and secondary. Whole lung lavage is currently the gold standard therapy for severe cases of pulmonary alveolar proteinosis. In Sri Lanka this is the first reported successful whole lung lavage for a patient with pulmonary alveolar proteinosis.

**Case presentation:**

We describe the case of a 15-year-old Sri Lankan girl who presented with symptoms of progressive shortness of breath and dry cough for 6 months’ duration. She had a history of exposure to silica in her household environment. High-resolution computed tomography revealed crazy paving appearance in both lungs suggestive of pulmonary alveolar proteinosis. An open lung biopsy revealed intra-alveolar granular amphophilic material which was strongly periodic acid–Schiff positive and diastase resistant, which is consistent with pulmonary alveolar proteinosis. She was followed up for 2 years with periodical segmental bronchoalveolar lavages which showed minimal improvement in her symptoms as well as in exercise desaturation. Due to severe dyspnea and hypoxemia on exertion, she underwent whole lung lavage. It resulted in a marked improvement in her symptoms, exercise desaturation, and chest X-ray results.

**Conclusion:**

Whole lung lavage was successfully performed for the first time in Sri Lanka for a patient with pulmonary alveolar proteinosis.

## Background

Pulmonary alveolar proteinosis (PAP) is a rare disease characterized by the accumulation of a lipoproteinaceous, eosinophilic, periodic acid–Schiff (PAS) positive material within the alveoli. The disease was first described by Rosen *et al.* in 1958 [1].

There are three different types of PAP: congenital PAP (2 % of total cases), secondary PAP (less than 10 % of total cases), and acquired or adult type PAP (90 % of cases), which is also referred to as primary or idiopathic. Primary acquired PAP occurs as a disorder of unknown etiology; it is not associated with any familial predisposition. The secondary form develops in adulthood and is found in association with high levels of dust exposure (for example silica, aluminum, titanium, indium-tin oxide) and hematological malignancies [2]. Macrophage dysfunction plays an important role in the initiation and propagation of the disease. The major symptoms are progressive dyspnea on exertion, cough, fatigue, weight loss, and low grade fever [3]. Whole lung lavage (WLL) should be considered in patients with deteriorating lung function and in cases with progressively worsening symptoms. It is still the most effective treatment for PAP [4].

In Sri Lanka there are only two previously reported cases of PAP who were managed with therapeutic-limited bronchoalveolar lavages (BALs) [5, 6]. We report the case of an adolescent girl with PAP who underwent therapeutic WLL, which was the first WLL in Sri Lanka.

## Case presentation

Our patient is a Sri Lankan girl; she is currently aged 17 years and has a body mass index (BMI) of 20.34 kg/m^2^. She was previously healthy. She has a significant history of exposure to silica from a stone breaking plant in the vicinity of her residence.

### Development of lung involvement and diagnosis of PAP

In 2014 at 15 years of age, she presented to our clinic with a 6-month history of progressive dyspnea which limited her exercise capacity. She noticed that she started becoming breathless while she walked from the school gate to the classroom carrying her school bag. She had a mild nonproductive cough, weight loss, and poor appetite in addition to breathlessness. She had no clubbing, cyanosis, or ankle edema and had no evidence of lymphadenopathy. She was not pale. A respiratory system examination revealed a respiratory rate of 28 breaths per minute but breath sounds were normal without any added sounds. Cardiovascular, abdominal, and central nervous system examinations were normal.

**Fig. 1:**
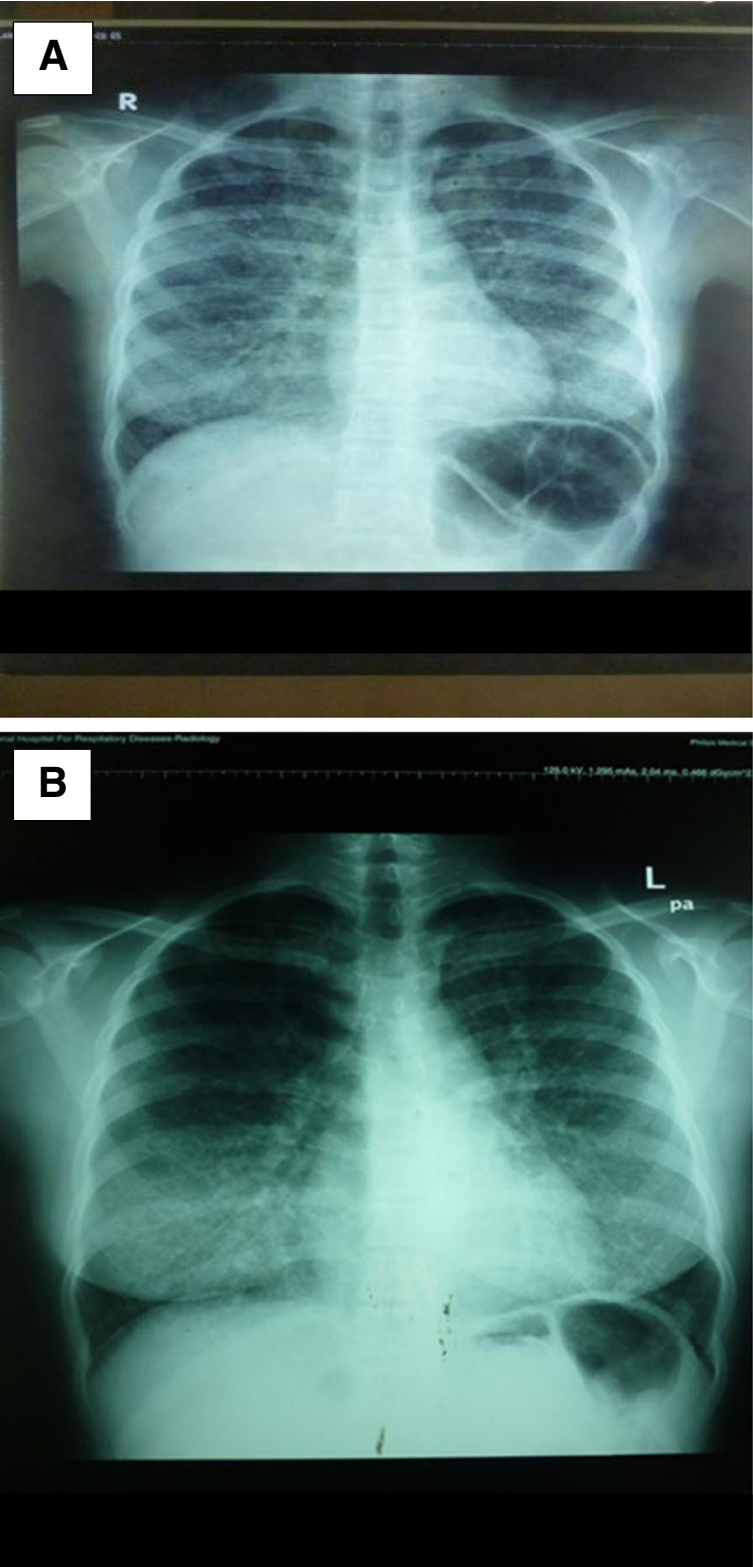
**a** Chest radiograph demonstrated reticular nodular pattern of bilateral perihilar distribution. **b** A repeated chest radiograph performed following whole lung lavages demonstrated an improvement in reticular nodular pattern

**Fig. 2:**
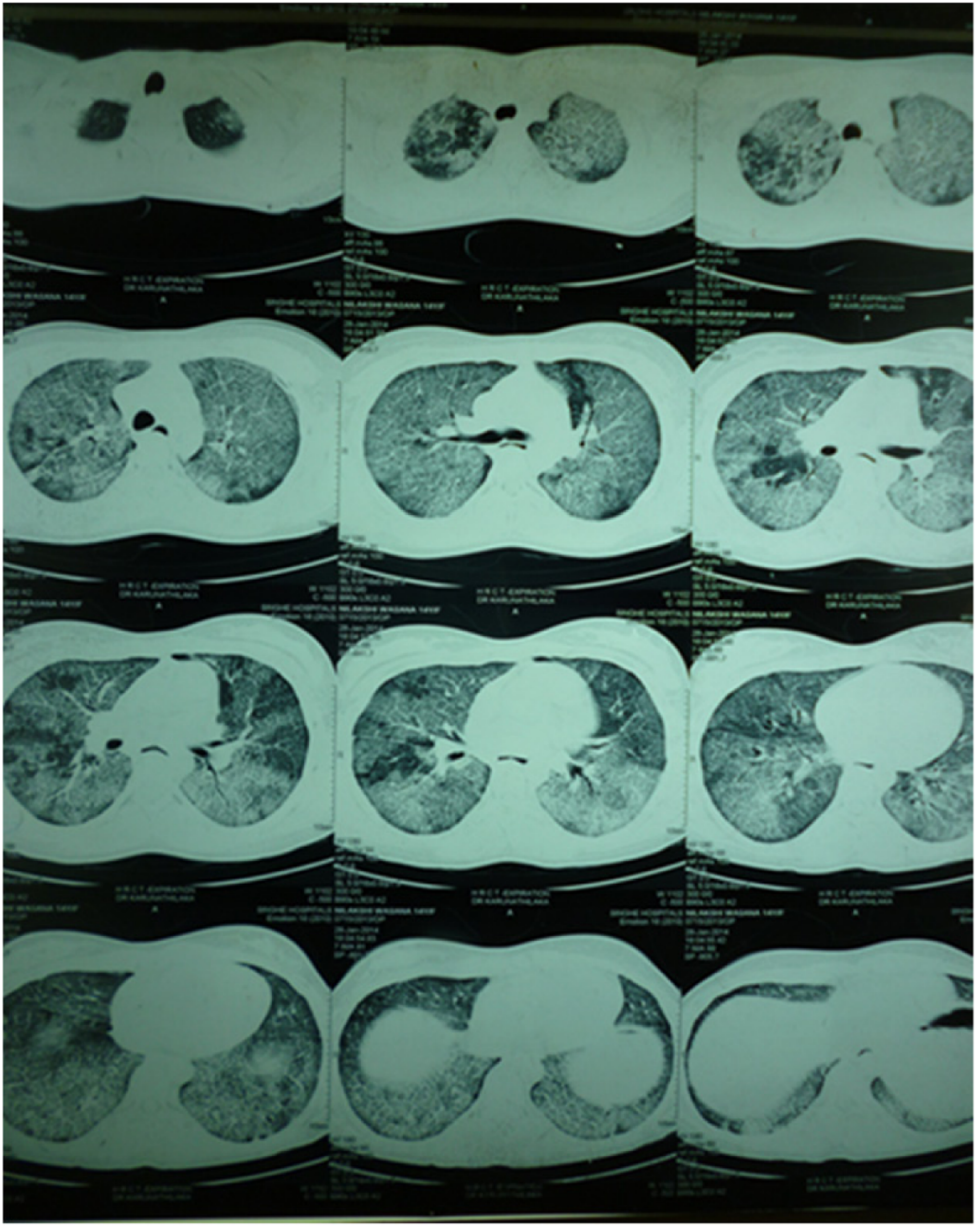
High-resolution computed tomography scan revealed diffuse crazy paving appearance in upper, mid, and lower lobes of both lungs with ground glass opacity and smooth interlobular septal thickening

Her complete blood count, erythrocyte sedimentation rate (ESR), and C-reactive protein (CRP) were normal. A chest X-ray revealed a reticular nodular pattern of bilateral perihilar distribution (Fig. 1a, b ). A two-dimensional echocardiogram showed mitral valve prolapse and tricuspid valve prolapse. Direct sputum smear microscopy for acid-fast bacilli was negative. A Gene Xpert test for *Mycobacterium tuberculosis* genome detection was negative. A high-resolution computed tomography (HRCT) scan revealed diffuse crazy paving appearance in upper, mid, and lower lobes of both lungs with ground glass opacity and smooth interlobular septal thickening (Fig. 2). A pulmonary function test revealed a restrictive pattern: forced vital capacity (FVC)=1.87 (69.5 %), forced expiratory volume in 1 second (FEV1)=1.84 (73.5 %), and FEV1/FVC=98.3 %.

**Fig. 3:**
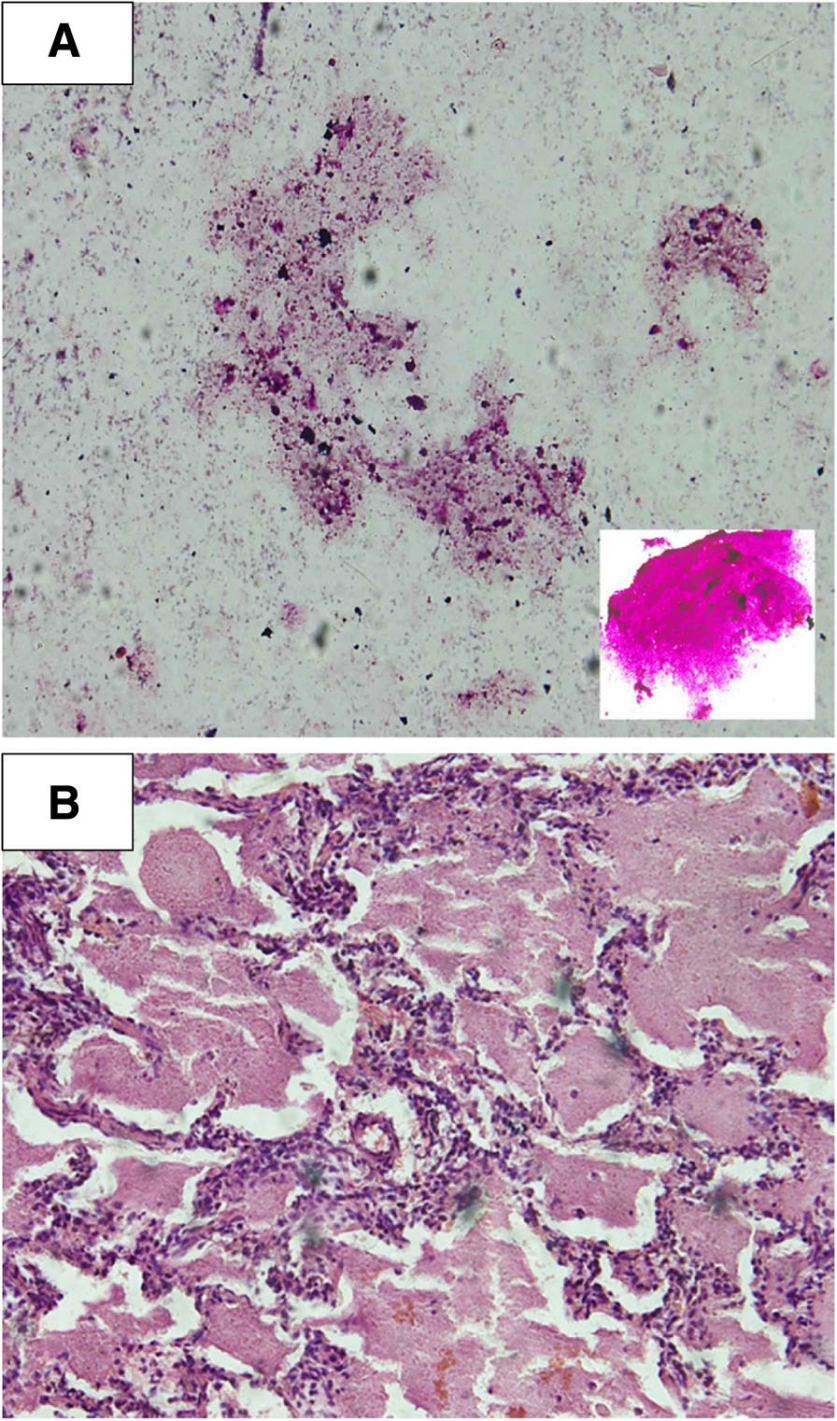
**a** Cytology smear of lavage material shows scattered pinky brown casts and granular material. Hematoxylin and eosin stain, 100× magnification. **Inset** Periodic acid–Schiff staining shows these casts to be periodic acid–Schiff positive. **b** Open lung biopsy showed preservation of the alveolar architecture, mild interstitial chronic inflammation cells, and filling of alveolar spaces with granular amphophilic material with a few histiocytes and pigment

A 6-minute walk test showed exercise desaturation from 92 % to 85 %. Her arterial blood gas (ABG) revealed significant hypoxemia while breathing room air: partial pressure of oxygen (PaO_2_) 58 mmHg. Her alveolar-arterial gradient was 45.6 mmHg. A bronchial wash for cytology showed PAS-positive material; bronchial brush cytology showed no malignant cells. Diastase staining of lavage material and biopsy revealed that the intra-alveolar material was PAS positive and diastase resistant (Fig. 3a). An open lung biopsy revealed that 60 % of the alveolar lumina contained granular amphophilic material with a few histiocytes and pigment material. The interstitium showed a mild increase in chronic inflammatory cells (Fig. 3b). Her lactate dehydrogenase (LDH) level was 535 U/L (140 to 280). Carbon monoxide diffusion capacity (DLCO) was not done prior to the intervention due to unavailability of resources during that time period. Her blood picture showed no morphological evidence of an hematological malignancy. A human immunodeficiency virus (HIV) antibody test was negative. A diagnosis of PAP was confirmed with the HRCT pattern, lung biopsy, and lavage fluid characteristics.

### Two-year follow up

Following diagnosis she was followed up at our respiratory out-patient clinic with monitoring of symptoms and pulmonary function tests on a regular basis. She showed progressive dyspnea and significant exercise desaturation during the first month of her follow up; so she underwent her first therapeutic BAL in March 2014. Segmental lavage of left upper, lower, and lingular lobes was performed under general anesthesia. She was monitored in our Intensive Care Unit (ICU) post-procedurally. She underwent seven episodes of therapeutic segmental BAL over a period of 1.5 years from March 2014 to August 2015. At each episode, she was carefully assessed for fitness for the procedure with the indication of exercise desaturation and progressive dyspnea. Although she had mild clinical improvement in her breathlessness, the improvement in exercise desaturation and radiological appearance was minimal, so we decided to proceed to total lung lavage under general anesthesia.

### WLL treatment

She underwent WLL in the operation theatre. Pulmonary function tests (spirometry, 6-minute walking distance, ABG analysis) and radiological assessment (chest X-ray) were carried out prior to the procedure. For WLL of her left lung, our patient underwent general anesthesia and was intubated with a double lumen endotracheal (ET) tube of 35 gauge. Tracheal and bronchial balloons were inflated to isolate her two lungs. The correct positioning of the ET tube was confirmed using a flexible bronchoscope. The lung isolation was reconfirmed following immersion of each end of the ET tube in water to look for air bubbling while the other lung was ventilated. Both lungs were ventilated for approximately 5 minutes to oxygenate our patient adequately before the procedure; then, single lung ventilation was commenced and was observed for several minutes to see whether appropriate oxygenation took place during single lung ventilation. After letting her left lung de-gas, warm saline (at 37 °C) run through a blood warmer, was instilled via the ET tube until it filled up to the visible level (mouth level) of the tube. During the first cycle a volume of 300 ml was instilled and chest percussion was performed by a physiotherapist for 5 minutes. Then the effluent was taken out by elevating the foot end of the bed and using a low pressure suction catheter. During this cycle around 200 ml of the milky effluent was removed. The procedure was repeated ten times using instillation of warm saline and removal of the effluent as described above. A total of 2300 ml of fluid was instilled. WLL was performed satisfactorily with the removal of an effluent of 1900 ml which became progressively less opaque. No complications were observed during or after WLL. In addition to using warm saline, a warming blanket was used to maintain the core temperature of our patient during the procedure.

**Fig. 4:**
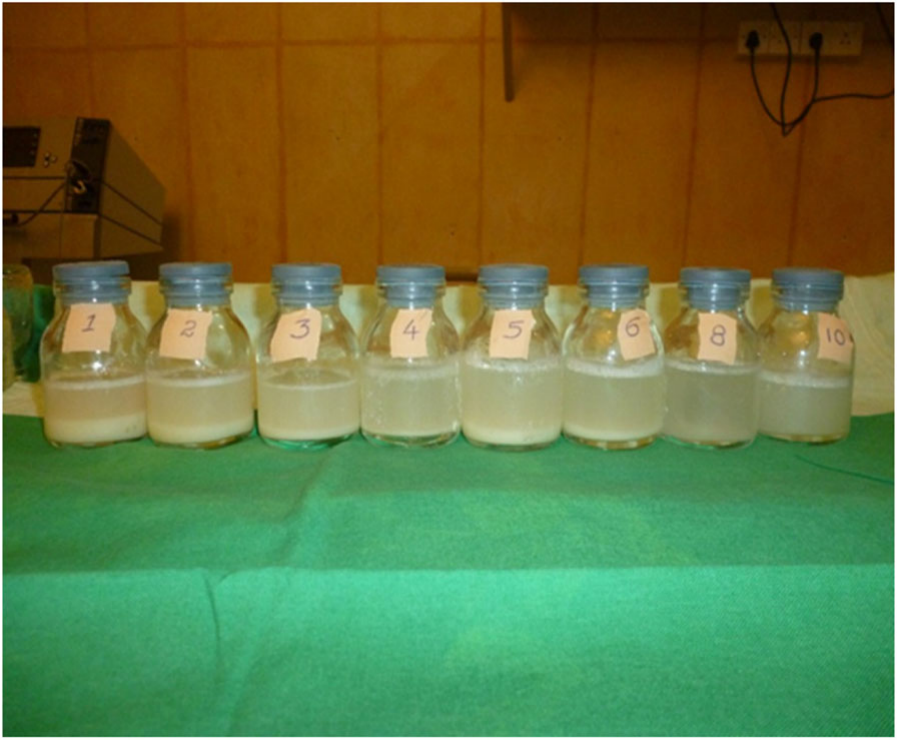
The effluent from whole lung lavage which gave an initial “milky” appearance with a high sediment level (Bottle 1) and gradually turned less opaque with a lower sediment level (Bottle 10). The number on each bottle corresponds to the number of the respective cycle of whole lung lavage

The milky effluent that came out initially produced a higher volume of sediment on standing (Fig. 4, Bottle 1). The height of the sediment reduced towards the last cycles of the procedure (Fig. 4, Bottles 8 and 10). Our patient was observed in the ICU for approximately 12 hours following the procedure and developed no complication. She was transferred to a ward following ICU observation and she was discharged on day 3 following the procedure. Sequential WLL was done on her right lung, 1 month after the left WLL. A chest X-ray, ABG analysis, 6-minute walk test, and spirometry were repeated pre-WLL and post-WLL (Table 1).

**Table 1:** Comparison of pulmonary function before and after the whole lung lavages

	Pre-whole lung lavages	Post-whole lung lavages
ABG	pH 7.39	pH 7.37
	pO_2_ 58	pO_2_ 87
	pCO_2_ 31	pCO_2_ 38
	HCO_3_– 20.3	HCO_3_– 22.6
Spirometry	FVC 1.87	FVC 2.09
	FEV1 1.84	FEV1 1.97
	Ratio 98.3 %	Ratio 95.6 %
DLCO	Not done	15.75 (78 %)
Six-minute walk test (exercise desaturation)	92 % → 84 %	96 % → 94 %
Alveolar-arterial gradient	45.6 mmHg	17.2 mmHg

*ABG* arterial blood gas, *DLCO* carbon monoxide diffusion capacity, *FEV1* forced expiratory volume in 1 second, *FVC* forced vital capacity, *HCO*_*3*_ *–* bicarbonate, *pCO*_*2*_ partial pressure of carbon dioxide, *PO*_*2*_ partial pressure of oxygen

## Discussion

The incidence of PAP is four times higher in males than in females and typically presents between 20 and 50 years of age [7]. Up to now, all the three cases found in Sri Lanka are of female gender, although our case is the only patient who presented in her adolescence. The commonest chest X-ray presentations in patients with PAP are bilateral symmetrical alveolar opacities located centrally in the mid and lower lung zones, often in a “batwing” distribution. The chest X-ray of our patient shows a reticular nodular pattern of bilateral perihilar distribution. The main features of PAP in HRCT were present in our patient, constituting thickened intralobular structures and interlobular septa, with no architectural distortion, often with polygonal shapes, sometimes called “crazy paving” pattern, and areas of ground glass opacification with a “geographic” pattern as the diseased lung is sharply demarcated from the surrounding normal lung tissue [8]. BAL findings are diagnostic in PAP [9]. It is opaque or milky in appearance due to the abundant lipoproteinaceous material, which may settle upon standing. Histology of lung tissue obtained via transbronchial biopsy or open lung biopsy is a useful adjunct in the final diagnosis [10] and our patient’s specimen showed characteristic pathological features. The commonest acquired type of PAP is associated with a high prevalence of anti-granulocyte-macrophage colony-stimulating factor (anti-GM-CSF) antibody. Several lines of evidence suggest that diminished anti-GM-CSF protein or function plays a key role in the pathogenesis of PAP and is responsible for the observed impairment in surfactant processing [11]. An anti-GM-CSF antibody test was not done since it was not available in the local setting.

Secondary PAP can be associated with three main clinical settings. First, infection of the lung, most commonly with *Nocardia asteroides*, TB (Tuberculosis), *Mycobacterium avium-intracellulare*, or *Pneumocystis carinii*. Second, hematologic malignancies and other conditions that alter the patient’s immune status, for example, lymphoma, leukemia, or AIDS (Acquired Immune Deficiency Syndrome). Third, exposure to inhaled chemicals and minerals, for example, fumes, dusts, silica [2], aluminum, insecticides, or titanium [12]. In our patient, infections and malignancies were excluded with investigations. Since she had a history of significant exposure to silica, she is most probably a case of secondary type of PAP caused by inhalation of silica dust. Silicoproteinosis usually manifests within 3 years of the initial exposure as rapidly progressive shortness of breath often associated with constitutional symptoms. The course of the disease is relentlessly progressive. Most of the reported cases have been fatal within months [2, 13]. Although 3 years elapsed following the symptom onset of our case, the disease progression is not as rapid as described in the literature.

WLL was first described by Ramirez and colleagues in 1965 and further modified by Wasserman and coworkers in 1968 [14]. For patients who have moderate to severe symptoms and hypoxemia, WLL under general anesthesia via a double-lumen ET tube is the most widely accepted and effective form of treatment [15]. Specific indications for lung lavage include a definitive histologic diagnosis and one of the following: resting PaO_2_ <65 mmHg (at sea level), alveolar-arterial O_2_ gradient ≥40 mmHg, measured shunt fraction >10 to 12 %, or severe dyspnea and hypoxemia at rest or on exercise. Our patient had most of the above indications for undergoing WLL.

The technique of WLL is well described [16]. In general, aliquots of 1 to 1.5 liters of warm saline are required for each lavage, and a total of approximately 10 to 15 lavages are used for clearing of the lavage effluent from each lung [17]. Chest percussion during the lavage procedure significantly increases the recovery of the lipoproteinaceous material [18]. Complications of WLL include malpositioning of the ET tube, saline spillover into the unlavaged ventilated lung, and hydropneumothorax. After WLL, symptoms often improve dramatically; however, long-term follow up is needed since the clinical outcome is variable. While only one lavage may be required for a prolonged remission, up to 55 % cases may need a repeated lavage at 6-month to 12-month intervals [3]. It is often impossible to perform therapeutic total lung lavage in most patients who are newly diagnosed as having PAP due to the above potential complications and because the patients are usually hypoxemic and in poor clinical condition. In such cases, multiple segmental or lobar lavages by fiberoptic bronchoscopy (FOB) have been reported as a possible alternative to WLL [19]. Although our patient underwent therapeutically limited BAL several times under general anesthesia in the operation theatre, she showed minimal improvement in her clinical, radiological, and pulmonary function. So we proceeded to WLL, which is the definitive treatment for PAP. The volume of warm saline needed to be used for the WLL was comparatively low in our patient. This is probably due to the small lung volume in an adolescent patient with a low BMI and chronic interstitial lung disease leading to loss of lung volume. Our patient showed marked improvement in her symptoms, pulmonary function, and X-ray results following sequential WLL of both lungs. However, long-term follow up is essential to assess the need for repeated WLL.

## Conclusion

In conclusion, therapeutic WLL can be successfully performed as a definitive treatment option for a patient with PAP in Sri Lanka.

## Abbreviations

ABG: Arterial blood gas
AIDS: Acquired Immune Deficiency Syndrome
BAL: Bronchoalveolar lavage
BMI: Body mass index
CRP: C-reactive protein
DLCO: Carbon monoxide diffusion capacity
ESR: Erythrocyte sedimentation rate
ET: Endotracheal
FEV1: Forced expiratory volume in 1 second
FOB: Fiberoptic bronchoscopy
FVC: Forced vital capacity
GM-CSF: Granulocyte-macrophage colony-stimulating factor
HIV: Human immunodeficiency virus
HRCT: High-resolution computed tomography
ICU: Intensive Care Unit
LDH: Lactate dehydrogenase
PaO_2_: Partial pressure of oxygen
PAP: Pulmonary alveolar proteinosis
PAS: Periodic acid–Schiff
TB: Tuberculosis
WLL: Whole lung lavage
